# Baby-Led Weaning: The Evidence to Date

**DOI:** 10.1007/s13668-017-0201-2

**Published:** 2017-04-29

**Authors:** Amy Brown, Sara Wyn Jones, Hannah Rowan

**Affiliations:** Department of Public Health, Policy and Social Sciences, Swansea University, 136 Haldane Building, Singleton Park, Swansea, SA2 8PP UK

**Keywords:** Baby-led weaning, Weaning, Introduction solid foods, Complementary feeding, Responsive feeding, Infant-led, Breastfeeding, Maternal, Infant, Weight, Eating behaviour, Appetite control, Maternal feeding style, Nutrient intake, Energy regulation, Choking

## Abstract

**Purpose of Review:**

Infants are traditionally introduced to solid foods using spoon-feeding of specially prepared infant foods.

**Recent Findings:**

However, over the last 10–15 years, an alternative approach termed ‘baby-led weaning’ has grown in popularity. This approach involves allowing infants to self-feed family foods, encouraging the infant to set the pace and intake of the meal. Proponents of the approach believe it promotes healthy eating behaviour and weight gain trajectories, and evidence is starting to build surrounding the method. This review brings together all empirical evidence to date examining behaviours associated with the approach, its outcomes and confounding factors.

**Summary:**

Overall, although there is limited evidence suggesting that a baby-led approach may encourage positive outcomes, limitations of the data leave these conclusions weak. Further research is needed, particularly to explore pathways to impact and understand the approach in different contexts and populations.

## Introduction

Over the last century, tradition has been to introduce infants to solid foods using spoon-feeding of specially prepared infant foods [1]. Current World Health Organisation guidance recommend that infants are initially offered smoothly blended foods, progressing in texture as the infant until by 12 months, infants should be eating family foods. Finger foods, e.g. whole foods, are recommended from 8 months, but alongside purees, rather than as the main diet [2]. This is echoed in national infant feeding guidance around the globe, although timing of finger foods vary. In the UK for example, the Department of Health recommend finger foods from the start of introducing complementary foods (https://www.nhs.uk/start4life/first-foods) whilst in New Zealand babies can be offered finger foods from 7 months(http://www.health.govt.nz/your-health/pregnancy-and-kids/first-year/6-12-months/feeding-your-baby).

However, over the last 10–15 years, an alternative approach known as ‘baby-led weaning’ has grown in popularity. Here, instead of blending special foods, infants are allowed to self-feed family foods in their whole form. The emphasis is on allowing infants to choose what, and how much, they eat and for the infant to be part of family mealtimes [3]. Although in reality this approach is likely what mothers did for millennia before the introduction of specially prepared foods, baby-led weaning represents an alternative to the modern, industry-driven infant feeding culture [4].

Part of this growth may be explained by the World Health Organisation raising the guidance in 2003 as to when infants should be introduced to solid foods to 6 months old [2]. Prior to this, infants were given solid foods at around 4 months but physiologically, the experience of introducing solids to a 4-month-old is very different to a 6-month-old. Naylor and Morrow examine how, at around 6 months, the majority of infants develop the skills needed to self-feed, including being able to sit up unsupported, bring food to their mouth, chew and swallow food [5]. Few infants at 4 months old would be able to do this; hence, pureeing and spoon-feeding was a necessity. This change however opened the opportunity for more infants to have solely whole foods from the start of weaning.

Tracing the emergence of baby-led weaning through initial discussion of the approach in publications and in online parenting forums, it is apparent that the method started to gain momentum from around 2001 [6]. Following the publication of Rapley’s global selling ‘baby-led weaning book’ in 2008 [3], growing numbers of families started to follow the approach. Estimates of current numbers following the approach are difficult to make, as no official population data has been collected on uptake of this style of complementary feeding. However, Google returns just under one million results for ‘baby-led weaning’ [accessed 08/12/2016] with a range of groups on social media.

Proponents of baby-led weaning have always suggested that the method offers a range of benefits to babies, from better appetite control, to a wider diet and even motor skill development. Initially these suggestions came from anecdotal experience in practice, followed by small-scale observational studies by Rapley herself for her MSc thesis [7]. Given (A) the established literature on the importance of responsive feeding [8], (B) understanding of the importance of early experiences on the development of long-term eating behaviour and weight gain trajectories [9] and (C) possible (albeit not conclusive) protection of delayed introduction of solids for a healthy weight, [10] it is logical that a method that encourages this may lead to healthier outcomes. However, to offer evidenced based guidelines, and provide professional support to new parents, an empirical evidence base must establish this, ideally distinguishing which elements of the approach are effective.

The aim of this review was therefore to establish the evidence underpinning the baby-led approach so far, identify limitations and explore the need for future research in this area.

### Search Methods

A review of the literature was performed, searching Web of Science, PubMed, Medline and Google Scholar for papers and theses of any date up to and including 05/12/2016. Search terms included baby-led weaning (including baby-led spellings), finger foods, self-feeding, family foods, responsive feeding and infant-led. To be included, papers had to (a) be original data as against commentary, (b) be published in English and (c) published in a developed nation to avoid ‘in order to discriminate from countries with a lower standard of living and poorer health status which would complicate the cross-country comparisons of infant feeding practice’ [11]. Postgraduate theses were included as many report findings from one ongoing key study.

### Types of Data Examined

Table 1 summarises the papers found. The majority of data clusters around two research groups in the UK and New Zealand. Two further studies were identified from the USA and Canada. All research papers were published between 2010 and 2016. The majority of papers identified used self-report questionnaire or interview data. One paper used existing cohort data to explore occurrence of self-feeding.

**Table 1:** Overview of study methodologies included in review

Authors	Type of data	Infant age range	Weaning type	Number of participants		Definition of baby-led weaning	Outcome measures
Brown and Lee [12 ^•^]	Maternal self-report questionnaire	6–12 months	Baby-led versus traditional	655 mothers		Proportion of pureed foods and spoon-feeding (BLW = 10% or less)	Comparison between BLW and traditional: demographic background of mother, timing of introduction to solid foods, type of first solids, weaning experience
Brown and Lee [13]	Maternal self-report questionnaire	6–12 months	Baby-led versus traditional	652 mothers		Proportion of pureed foods and spoon-feeding (BLW = 10% or less)	Maternal child-feeding style
Brown and Lee [14]	Maternal interview	12–18 months	Baby-led only	36 mothers		Self-defined	Experience of following a baby-led approach. Perceived benefits and negatives
Rowan and Harris [15]	Two 3-day diet diaries	6–9 months	Baby-led only	10 parents		Self-defined, parents required to read Rapley BLW book	Changes, if any, in parental diet after implementing BLW; description of foods offered to infants; similarity in parental and infant diets
Cameron et al. [16]	Maternal self-report questionnaire	6–12 months	Baby-led versus traditional	199 mothers		Adherent BLW: infant mostly/always self-fed; self-identified BLW <50% spoon-feeding; parent-led >50% spoon-feeding	Comparison between weaning groups: demographic information; parental attitudes and experiences of complementary feeding; types of foods offered
Cameron et al. [16]	Practitioner and maternal interviews	8–24 months	Baby-led only	31 Healthcare professionals; 20 mothers		Self-defined	Knowledge, attitudes and perceived benefits and concerns of healthcare professionals; experiences of mothers using BLW
Townsend and Pitchford [17]	Parental self-report questionnaire	20–78 months	Baby-led versus traditional	155 parents		Self-defined	Comparison between BLW and traditional: demographic background; infant feeding, weaning style; child’s food preferences and exposure; BMI
Arden and Abbott [18]	Maternal self-report questionnaire	9–15 months	Baby-led only	15 parents		Self-defined	Experience of following a baby-led approach; perceived benefits and practical issues; timing of introduction to solid foods
Brown and Lee [19]	Maternal self-report questionnaire	18–24 months	Baby-led versus traditional	298 mothers		Proportion of pureed foods and spoon-feeding (BLW = 10% or less)	Maternal demographic information; maternal feeding style; child eating behaviour; child and maternal BMI
Brown [20 ^•^]	Maternal self-report questionnaire	6–12 months	Baby-led versus traditional	604 mothers		Proportion of pureed foods and spoon-feeding (BLW = 10% or less)	Comparison between BLW and traditional: demographic background; maternal BMI; weaning style; maternal eating behaviour and personality
D’Andrea et al. 2016 [21]	Healthcare providers and maternal self-report questionnaires	Not stated	Baby-led only	33 Healthcare providers; 65 mothers		Self-defined	Knowledge, attitudes and perceived benefits and concerns of healthcare professionals; experiences and opinions of mothers using BLW
			BLISS study papers				
Cameron, et al. [22]	Parental interviews and 3-day diet diary or iron questionnaire		<6–9 months	Baby-led and BLISS only	23 infants 9 BLW 14 BLISS	Parents chose self-defined BLW or BLISS (modified BLW) group	Comparison between self-defined BLW and BLISS: maternal demographic information, high choking risk foods, high energy foods, iron containing foods
Erickson [23 ^••^]	Infant feeding questionnaire and weighed diet record		7 months	Baby-led (BLISS) versus traditional	202 infants 101 BLISS (BLW) 101 traditional	Randomised to either BLISS (BLW) or control (traditional)	Adherence to BLISS protocol, food group intake, nutrient intake
Morison et al. [24]	Parental feeding questionnaire and weighed diet record		6–8 months	Baby-led versus traditional	51 infants 18 full BLW 7 partial BLW 26 traditional	Self-defined	Comparison between BLW and traditional: food, nutrient and family meal intakes, feeding behaviours
Fangupo et al. [25]	Maternal report in five questionnaires		0–12 months	Baby-led (BLISS) versus traditional	206 infants 105 BLISS (BLW)—95 completed 101 traditional—89 completed	Randomised to either BLISS (BLW) or control (traditional)	Comparison between BLW and traditional: gagging and choking incidents, types of foods offered
			Papers not directly investigating BLW				
Arden [26]	Maternal online questionnaire		6 months	All	105 mothers	Self-defined	Factors associated with timing of weaning Maternal demographic information
Wright et al. [27]	Maternal self-report questionnaire and finger feeding diary		4–>8 months	All	510 infants	Not studied directly	Demographic and developmental information, timing of first reaching out for food, timing of first finger foods
Moore et al. [28]	Parental online questionnaire		N/A, babies had been weaned	All	3607 parents	Self-defined	Factors associated with timing of weaning

Five papers were identified relating to a single randomised controlled trial which examined outcomes of the method. The Baby-Led Introduction to SolidS (BLISS) trial allocated mothers to either standard care or modified baby-led approach. Those in the modified group were encouraged to allow their baby to self-feed but were educated as to the importance of providing iron-rich and energy-dense foods and foods that posed a low choking risk. The primary outcome of the study is the impact of approach upon BMI at 12 months, but other outcome measures include measurements of diet quality, energy and nutrient intake, developmental skills, choking risk and parental acceptability of BLW as an approach. This trial is ongoing and these data are being collected by anthropometric measures, questionnaires and blood serum testing [28].

From these data, several key data themes emerged:

## Is It a Feasible Approach?

Baby-led weaning requires infants to be able to self-feed successfully enough to maintain sufficient energy and nutrient intake. Although Naylor and Morrow [5] present a rationale for infants being able to self-feed at around 6 months of age, there is little data examining ability to do so. It should however be noted that no empirical data exists on efficacy of spoon-feeding and the UK Department of Health do recommend finger foods from the start of weaning [3].

To examine this, one paper used data from the Gateshead Millennium study cohort to retrospectively examine the percentage of infants who were reported to have grasped food with their hands. For those aged 4–6 months, 68% had reached out for food, 85% at 6–7 months and 96% at 7–8 months. However, the authors note that this may be an underestimation as it relied on infants being offered the opportunity. Conversely, it may represent an overestimation if parents’ judgements of ‘readiness’ are incorrect, especially at the younger age range of 4–6 months. However, it is likely that if parents were unsure of whether they should give finger foods, were anxious about suitability or simply did not want to, the infant would not have been given the opportunity. The paper concluded that most infants should be capable of self-feeding by around 6 months, although this may be delayed amongst children with failure to thrive, feeding difficulties or sensory sensitivity [27].

## How do Baby-Led and Traditional Methods Differ?

### Timing of Introduction to Solids

Following a baby-led approach is associated with a later introduction of solid foods, e.g. [12 ^•^, 26, 29]. It is unclear as to whether those who choose to follow the method would have naturally delayed introduction, or whether choosing to follow the method necessitates a later introduction due to physiological readiness. However, in the BLISS trial, two thirds of those assigned to the baby-led group delayed introduction until 6 months old, compared to only 18% of the usual care group, suggesting that a decision (or direction) to follow the approach may lead to delayed introduction [23 ^••^]. As a very early introduction to solids (before 4 months) is a possible risk factor for overweight, with a weaker suggestion that introduction before 6 months may also increase risk [10], any study examining outcome of weaning approach must control for timing of introduction.

### First Foods

Baby-led infants are more likely to have had a whole food at their first food and for this to be a food from the family diet [12 ^•^, 16]. If the first food was a pureed food, it was more likely to be home made rather than a product purchased. Baby-led infants were significantly less likely to be given baby rice [12 ^•^].

### Milk Feeding

Mothers who follow a baby-led approach are more likely to have initiated breastfeeding at birth, continued breastfeeding for longer and to currently be breastfeeding at the time of study [12 ^•^, 17]. Potentially, mothers who breastfeed may be more likely to baby-led wean. However, when randomised in the BLISS trial, the baby-led group breastfed for a mean duration of 21.7 weeks compared to 17.3 weeks for those in the standard group, suggesting a baby-led approach may be protective or encouraging of breastfeeding [23 ^••^]. However, baby-led weaning is not exclusively associated with breastfeeding; mothers who formula feed also follow the approach [3, 6, 30]. Breastfeeding mothers are more likely to adopt a responsive feeding style during infancy [31], during weaning [13] and as toddlers [32]. Moreover, a longer breastfeeding duration has been associated with lower risk of overweight [33], reduced fussiness [34], a slower rate of eating [35] and better appetite control [14]. It is therefore important that breastfeeding duration is controlled for in research examining its outcomes, due to both its effect and association with a generalised responsive feeding styles (see next section).

### A Responsive Feeding Style

A central element of the baby-led approach is allowing the infant to self-feed. This would naturally encourage a responsive maternal feeding style low in control. This was confirmed in one longitudinal study that used a modified version of the child-feeding questionnaire at 6–12 [36] and 18–24 [19] months to measure maternal pressure to eat, restriction, concern for child weight and monitoring. At both stage, mothers who followed a baby-led approach were lower in each of these, adopting a more responsive feeding style, compared to those following a traditional approach.

## Is there a Difference in Diet Consumed?

Data concerning actual nutrient intake according to weaning approach is sparse. This may be in part due to the particular difficulty in accurately measuring baby’s intake in baby-led weaning because self-feeding is an exploratory, less contained and often messy process. However, the perceived impact of weaning approach upon nutrients consumed is a key theme throughout interviews with parents and practitioners.

Notably, parents typically perceive the baby-led approach to have a positive impact upon diet, whilst practitioners perceive it as a risk. For example, in qualitative research exploring the perceived benefits of a baby-led approach amongst mothers who followed the method, all four papers [18, 21, 37, 38] highlight that mothers believe the method to increase variety of foods and nutrients consumed. Meanwhile, Brown and Lee [37] report that mothers who followed baby-led weaning noted that their health professional was against the method or did not understand it, meaning that they were unable to get professional advice from their health visitor. In a Canadian study, less than half of health professionals (48.5%) noted that they were prepared to support their method in their own practice, because of their concerns around choking, growth faltering and iron intake. This was compared to all mothers being happy to recommend the approach to others [21]. Similar results emerged from a study exploring health practitioners’ perceptions of baby-led weaning in New Zealand. Although benefits were noted, practitioners held concerns around faltering growth due to low energy density foods being offered and potential lack of early self-feeding skill [38].

### Foods Offered

Two studies have examined whether infants are offered family foods or those made specifically for infants. As noted above, infants following a baby-led diet are reported to be more likely to eat family foods rather than specially prepared infant foods and join in family meal times [12 ^••^, 16]. However, one study exploring the diets of families following a baby-led approach found that infants were offered only 57% of the same food as their parents, although ate with their parents on 85% of occasions [15].

Consuming family foods may carry the risk that infants consume foods that are unsuitable, e.g. being too high in sodium content or a choking hazard. However, in interviews with mothers who had followed a baby-led approach, Brown and Lee [37] found that mothers reported modifying their family diet to suit the baby-led infant. Conversely, a small-scale pilot study of families following the baby-led weaning approach compared family food intake pre- and post-weaning to explore whether parents diet (and therefore food offered to the infant) changed in macro- and micronutrient content, but found that there was no significant change. Moreover, analysis of diet diaries found that adults’ diets were higher in sodium and saturated fat content than both UK and USA guidelines [15]. Potentially, infants may be exposed to nutrients that are less suitable than typical pre-prepared weaning foods, but further research is needed to establish this.

### Nutrient Intake

In terms of actual nutrient intake, only the BLISS study has examined this [28]. Using a 3-day weighed food record, the nutrients consumed were compared between the two groups for infants ages 7 months (*n* = 147; BLISS *n* = 76, control *n* = 71). Infants in the BLISS group were more likely to consume ‘meat’, ‘cow’s milk or dairy products’ and ‘sweet foods’ but were, surprisingly, also more likely to consume ‘powdered infant cereal’. Meanwhile, the usual care group were significantly more likely to eat ‘ready-to-eat commercial infant foods’. No differences were found between the groups for consumption of ‘fruit and fruit juice’, ‘vegetables’ and ‘bread, pasta, rice and low sugar cereals’ [23 ^••^].

For overall macronutrient intake, there were no significant differences between the groups. Infants from both groups consumed 10% of energy intake from protein, 45% from fat and 45% from carbohydrates. However, when data was analysed excluding milk feeds, infants in the BLISS group consumed significantly more protein and fat. This may be because the inclusion of protein-rich food at every meal was encouraged as part of the BLISS intervention. For mineral intake, infants in the BLISS group consumed higher levels of sodium, selenium and lower levels of vitamin A. Infants in the BLISS group consumed more than double that of the usual care group [23 ^••^]. However, it is possible that the baby-led data is less accurate given the difficulty of measuring actual consumption (versus amount squashed, dropped and smeared), although this is also an inherent issue to spoon-feeding which is not necessarily neat.

Low iron consumption is often cited as a concern for infants being introduced to solid foods. In a pilot of the BLISS trial, mothers who were planning to follow a baby-led approach were assigned to simply follow their own version of BLW, or to the BLISS group with its emphasis on iron-rich and energy-dense foods, such as red meat or an iron-fortified infant cereal. Compared to the BLW group, the BLISS group had a higher introduction of iron-containing foods in the first week of introduction of solid foods, and offered more portions of such food at 6 months (2.4 versus 0.8 portions a day) [22].

### Energy Intake

Again, only the BLISS trial has examined energy intake through using food diaries to calculate infant intake. Comparison of intake at 7 months [23 ^••^] and 12 months [24] found no significant difference in energy intake between the two groups.

## Is there an Impact on Eating Behaviour?

In the interview studies with parents and practitioners discussed above, a similar pattern emerged as for nutrient intake as to how the baby-led approach was perceived to impact future eating behaviour. Mothers who followed the approach perceive that it promotes positive eating behaviour for infants, in terms of reduced fussiness and better appetite control [18, 21, 37, 38]. Conversely, although professionals believe that the approach might promote healthier eating behaviours, they typically add a proviso regarding concern for sufficient intake [21, 38].

### Food Preferences

One longitudinal study has examined perceived fussiness of infants weaned using traditional or baby-led approaches using the Child Eating Behaviour Questionnaire [39]. This found that infants who had followed a baby-led approach were less likely to be rated by their mother as fussy eaters at 18–24 months. However, when maternal child-feeding style (e.g. responsive feeding) was taken into account, the difference was no longer significant [19]. The data also relied on maternal self-report.

Two studies have explored food preferences amongst infants who have followed a baby-led approach. One examined reported differences in food preference at 12 months old, finding no difference between baby-led and traditional weaned groups [24]. In another study, the food preferences of preschool children who had been weaned using a baby-led or traditional approach were examined through parental self-report. Infants weaned using a baby-led approach had a preference for carbohydrates whilst those in the traditional group had a preference for sweet foods [17]. However, this study relied on retrospective recall of weaning approach over a 2 to 3-year period. Differences were also only marginally significant.

### Eating Behaviour

The longitudinal study above also examined maternal reported infant food responsivity (e.g. eating in response to food being present) and satiety responsivenes [39] (e.g. ability to stop eating when full). The study found that those who had followed a baby-led approach were significantly more likely to be rated at 18–24 months as less food responsive and more satiety responsive, suggesting better appetite control [19]. However, again it must be noted that this data was based on self-report. Longitudinal research is needed to follow up the longer-term trajectory of infants based on early weaning styles.

## Is there an Impact upon Weight?

In the longitudinal study stated above, no differences in weight were seen at birth or 6 months. However, infants who followed a traditional weaning approach were significantly heavier at 18–24 months compared to those following a baby-led approach, independently of birth weight, maternal weight, breastfeeding duration and maternal child-feeding style. Mean weight, in kilogrammes, of infants in the SW group was 12.86 (SD 3.73) compared with 11.79 (SD 3.53) in the BLW group. A difference in weight category was also seen. For those who had followed a baby-led approach, 86.5% were of normal weight, 8.1% overweight and 5.4% underweight. In comparison, 78.3% of those who had followed a traditional approach were normal weight, 19.2% overweight and 2.5% underweight. A significant difference was found for overweight between the two groups [19]. However, all weights were self-reported and the weaning group was self-selected leading to possible external factors playing a role.

Weight data was also collected in the study above that explored preschool children’s diet according to weaning approach. Overall, mean weight of those who had followed a baby-led approach was significantly less than those who had followed a traditional approach. In fact, the incidence of underweight and overweight (as against obesity) was higher amongst the BLW group, although numbers who fell into this category were very small. The majority of children were a healthy weight (BLW 81% and traditional 84%) [17]. However, a number of participants self-reported infant weight in this study and breastfeeding duration was not controlled for.

The BLISS trial used anthropometric measurements at 6, 7, 8, 9 and 12 months to compare differences between the two weaning groups. No child experienced growth faltering, but 32 infants experienced non-clinical slow growth. However, this was not significantly different between the two groups. It is important to note that the BLISS study participants were encouraged to include a high-energy food at each meal, which may have attenuated the risk of growth faltering. No data on wider weight measures has yet been reported [25].

## Is the Approach Safe?

Both the New Zealand and Canadian studies exploring health professionals’ attitudes towards baby-led weaning highlighted concerns regarding perceived choking risk. Work with parents reflects a different attitude. Parents following the method either report no concerns about choking [38] or those who were wary of choking at the beginning of the process reported becoming more relaxed and confident with experience [37].

In terms of choking frequency, this is a difficult behaviour to measure accurately. Data has to rely on self-identification of choking (as against gagging) and self-report. One study examining rates in New Zealand found no difference in choking incidences between groups [16], which was supported in the larger-scale BLISS trial work [25]. However, the BLISS data noted that choking occurred at least once in all weaning groups at a rate of 35% of infants. At 7 months, 52% had been offered foods that posed a choking risk and 95% at 12 months. However, there was no difference in this between weaning groups. Foods that were commonly choked on in both these studies included apple slices, crackers and sausages.

## What are the Limitations of the Data?

### Self-Report

As noted, a key limitation is that much of the data described above is based on self-report, or perceptions of mothers who have followed the method. Data may be inaccurate (e.g. weight) or there may be reporting bias given that mothers are supporters amongst a method that has received criticism from others. Food diaries can also be open to inaccuracies and socially desirable reporting.

### Self Selection

The majority of studies that examine differences in mothers who follow a baby-led versus traditional approach tend to find that mothers who follow a baby-led approach have a higher level of education and/or age compared to those following a traditional approach [12, 38], although this is not conclusive across all studies.

There are also differences outside of demographic background that may affect outcomes. One study showed that following a baby-led approach was associated with lower maternal restrained eating and lower anxiety [20^•^]—factors that are associated with a positive eating style and lower likelihood of overweight in older children. Higher maternal restraint is associated with increased child overeating [40] and child overweight [41] although not every study is conclusive [42]. Meanwhile, anxiety is associated with a more controlling feeding style [43]. Maternal wider anxiety, eating behaviour and control may affect willingness to allow self-feeding and regulation or raise concerns over choking risk, ruling out the option of a baby-led approach. It is important that further research considers wider parental interactions outside of weaning approach.

Furthermore, it is also possible that outcomes are based on infants’ temperaments who suited a baby-led approach. It is likely that less ‘settled’ babies are less likely to follow a baby-led approach, given in part that babies who are perceived as less settled, i.e. crying, wakeful at night, are more likely to have early solids [44]. Likewise, infants who are perceived as over- or underweight are more likely to receive solid foods early [45], which in turn is associated with risk of later overweight [10]. Mothers of these infants are unlikely to follow a baby-led approach, but these infants are also more likely to have more negative long-term eating difficulties. It should be noted here then that babies’ baseline temperament should be considered as a factor in determining early and long-term eating behaviours, both in and of itself, and in relation to the domino effect on feeding style and maternal level of control.

Given these issues, the question also therefore arises as to whether parents can be randomised to follow the method. In the BLISS study, where mothers were randomised to either a baby-led or standard care approach, adherence to approach was monitored. At 7 months, 64% of the baby-led group were classified as ‘adherent’ to the principles of BLW. In comparison, 11% of the standard care group had adopted a baby-led approach [23 ^••^]. The 36% that were classified as non-adherent in the baby-led group followed an approach that was mostly baby-led but did not stick to all elements rigidly, e.g. some parental feeding or altered textures to foods. However, this occurred at a much lower level than those in the usual care group.

### Definition of Baby-Led Weaning Used

Finally, the definition of a baby-led approach is itself not clear. Different research studies have approached the classification in different ways. Some have simply asked parents whether they self define as following the approach. Data from two studies has suggested this may not be accurate. In the Canadian study, some mothers who identified as BLW still offered pureed foods [21] whereas in New Zealand [16], 21% of mothers who identified as baby-led were not ‘adherent’, e.g. they used purees and spoon-feeding. Conversely, one group of studies has asked parents how frequently they use spoon-feeding and puree use, using a cut off of 10% or less of the time in both categories to define as baby-led [12 ^•^, 13, 19]. Consensus of a research definition would be useful, or indeed research to examine what degree of spoon or puree feeding has an impact, or whether it is indeed self-feeding that is the causal pathway (as against responsive feeding itself for example).

## What do We Now Need to Know?

Ultimately, we know that many of the key tenets of a baby-led approach, e.g. delayed weaning, responsive feeding and exposure to a range of foods, are important building blocks of healthy eating behaviour and weight gain trajectories. Indeed, responsive feeding is one of the key approaches to developing a healthy eating behaviour in later life [9] and is recognised as a critical element of the introduction of solid foods by the World Health Organisation [46]. However, at present, it is unclear as to whether a baby-led approach affects outcomes independently of these factors. Indeed, Sachs [47] questions whether the baby-led approach deserves recognition as a defining approach. Potentially, all that differs in terms of the baby-led approach and weaning guidelines is the absence of any spoon-feeding or pureeing of food. Further research is needed to unpick the most important elements of the approach in order to enable clearer advice to be given to new parents.

## Conclusions

Despite significant numbers of interest in the baby-led weaning approach, further evidence is still needed to explore its potential impact upon nutrient and energy intake, and as a consequence child weight and eating behaviour. Initial research, particularly that exploring the experiences of those who have successfully followed it, has suggested that the approach may foster the development of positive eating behaviour and potentially weight gain, but further large-scale rigorous is now needed to understand this. It is also critical that we understand the baby-led approach in context, across different populations and interpretations of the approach.

## References

[1] Jones S (2016). A history of baby-led weaning: the evolution of complementary feeding trends. Journal of Health Visiting..

[2] World Health Organization, UNICEF. Global strategy for infant and young child feeding. World Health Organization; 2003.

[3] • Rapley G, Murkett T. Baby-led weaning: Helping your baby to love good food. Random House; 2008. **This book overviews the baby-led weaning approach and examines its development. It provides an excellent overview and is the key text that many parents read*****.***

[4] Palmer G. Complementary feeding: nutrition, culture and politics. Pinter & Martin Publishers; 2011.

[5] • Naylor, A.; Morrow, A. Developmental readiness of normal full term infants to progress from exclusive breastfeeding to the introduction of complementary foods: reviews of the relevant literature concerning infant immunologic, gastrointestinal, oral motor and maternal reproductive and lactational development. Academy for Educational Development: Washington DC, USA, 2001. **This is a review paper examining the critical issue of developmental readiness for solid foods which examines the match between infants external ability to self-feed and internal readiness to digest solid foods*****.***

[6] Rapley G (2015). Baby-led weaning: the theory and evidence behind the approach. Journal of Health Visiting.

[7] Rapley G (2003). Can babies initiate and direct the weaning process? (Interprofessional health and community studies; care of the breastfeeding mother and child MSc).

[8] Ventura AK, Birch LL (2008). Does parenting affect children’s eating and weight status?. Int J Behav Nutr Phys Act.

[9] Schwartz C, Scholtens PA, Lalanne A, Weenen H, Nicklaus S (2011). Development of healthy eating habits early in life. Review of recent evidence and selected guidelines. Appetite.

[10] Moorcroft KE, Marshall JL, McCormick FM (2011). Association between timing of introducing solid foods and obesity in infancy and childhood: a systematic review. Maternal & child nutrition..

[11] •• Cameron SL, Heath AL, Taylor RW. How feasible is baby-led weaning as an approach to infant feeding? A review of the evidence. Nutrients. 2012;(4, 11):1575–609. **This review papers examines evidence underpinning the different elements of a baby-led approach, e.g. ability to self-feed, delaying solids until 6 months and exclusive breastfeeding until 6 months**10.3390/nu4111575 PMC3509508 23201835

[12] Brown A, Lee M (2011). A descriptive study investigating the use and nature of baby-led weaning in a UK sample of mothers. Maternal & child nutrition.

[13] Brown A, Lee M (2011). Maternal child-feeding style during the weaning period: association with infant weight and maternal eating style. Eat Behav.

[14] Brown A, Lee M (2012). Breastfeeding during the first year promotes satiety responsiveness in children aged 18–24 months. Pediatric obesity.

[15] Rowan H, Harris C (2012). Baby-led weaning and the family diet. A pilot study. Appetite.

[16] Cameron SL, Taylor RW, Heath AL (2013). Parent-led or baby-led? Associations between complementary feeding practices and health-related behaviours in a survey of New Zealand families. BMJ Open.

[17] Townsend E, Pitchford NJ (2012). Baby knows best? The impact of weaning style on food preferences and body mass index in early childhood in a case–controlled sample. BMJ Open.

[18] Arden MA, Abbott RL (2015). Experiences of baby-led weaning: trust, control and renegotiation. Maternal & child nutrition..

[19] Brown A, Lee MD (2015). Early influences on child satiety-responsiveness: the role of weaning style. Pediatric obesity..

[20] • Brown A. Differences in eating behaviour, well-being and personality between mothers following baby-led vs. traditional weaning styles. Matern Child Nutr. 2015. **This paper highlights that mothers who choose to adopt a baby-led approach may be different in background to those adopting a traditional approach. This difference in background might affect outcomes, indicating it is important to consider maternal background and wider feeding approach when examining the impact of a baby-led style.** 10.1111/mcn.12172 PMC6860066 25623385

[21] D’Andrea E, Jenkins K, Mathews M, Roebothan B. Baby-led weaning: a preliminary investigation. Can J Diet Pract Res. 2016;77(2):72–7. 10.3148/cjdpr-2015-045 26771760

[22] Cameron SL, Taylor RW, Heath AL (2015). Development and pilot testing of baby-led introduction to SolidS-a version of baby-led weaning modified to address concerns about iron deficiency, growth faltering and choking. BMC Pediatr.

[23] •• Erickson LW. A Baby-Led approach to complementary feeding: adherence and infant food and nutrient intakes at seven months of age (Doctoral dissertation, University of Otago) (2015). **This paper examines initial findings of the first randomized controlled trial that randomized parents to a baby-led or usual care approach. It examines adherence to group and intake of infants according to group**.

[24] Morison BJ, Taylor RW, Haszard JJ, Schramm CJ, Erickson LW, Fangupo LJ, Fleming EA, Luciano A, Heath AL (2016). How different are baby-led weaning and conventional complementary feeding? A cross-sectional study of infants aged 6–8 months. BMJ Open.

[25] Fangupo LJ, Heath AL, Williams SM, Williams LW, Morison BJ, Fleming EA, Taylor BJ, Wheeler BJ, Taylor RW (2016). A baby-led approach to eating solids and risk of choking. Pediatrics.

[26] Arden MA (2010). Conflicting influences on UK mothers’ decisions to introduce solid foods to their infants. Maternal & child nutrition..

[27] Wright CM, Cameron K, Tsiaka M, Parkinson KN (2011). Is baby-led weaning feasible? When do babies first reach out for and eat finger foods?. Maternal & child nutrition..

[28] Daniels L, Heath AL, Williams SM, Cameron SL, Fleming EA, Taylor BJ, Wheeler BJ, Gibson RS, Taylor RW (2015). Baby-Led Introduction to SolidS (BLISS) study: a randomised controlled trial of a baby-led approach to complementary feeding. BMC Pediatr.

[29] Moore, A.P.; Milligan, P.; Goff, L.M. An online survey of knowledge of the weaning guidelines, advice from health visitors and other factors that influence weaning timing in UK mothers. Matern. Child Nutr*.* 2012 10.1111/j.1740-8709.2012.00424.x PMC6860354 22708552

[30] Rapley G, Forste R, Cameron S, Brown A, Wright C. Baby-led weaning a new frontier? ICAN: Infant Child Adolesc Nutr. 2015:1941406415575931.

[31] Brown A, Lee M (2013). Breastfeeding is associated with a maternal feeding style low in control from birth. PLoS One.

[32] Farrow C, Blissett J (2006). Breast-feeding, maternal feeding practices and mealtime negativity at one year. Appetite.

[33] Amir LH, Donath S (2007). A systematic review of maternal obesity and breastfeeding intention, initiation and duration. BMC pregnancy and childbirth.

[34] Shim JE, Kim J, Mathai RA, STRONG Kids Research Team (2011). Associations of infant feeding practices and picky eating behaviors of preschool children. J Am Diet Assoc.

[35] Rogers SL, Blissett J (2017). Breastfeeding duration and its relation to weight gain, eating behaviours and positive maternal feeding practices in infancy. Appetite.

[36] Brown A, Lee M (2011). Maternal control of child feeding during the weaning period: differences between mothers following a baby-led or standard weaning approach. Matern Child Health J.

[37] Brown A, Lee M (2013). An exploration of experiences of mothers following a baby-led weaning style: developmental readiness for complementary foods. Maternal & child nutrition..

[38] Cameron SL, Heath AL, Taylor RW (2012). Healthcare professionals’ and mothers’ knowledge of, attitudes to and experiences with, baby-led weaning: a content analysis study. BMJ Open.

[39] Carnell S, Wardle J (2007). Measuring behavioural susceptibility to obesity: validation of the child eating behaviour questionnaire. Appetite.

[40] Rodgers RF, Paxton SJ, Massey R, Campbell KJ, Wertheim EH, Skouteris H, Gibbons K (2013). Maternal feeding practices predict weight gain and obesogenic eating behaviors in young children: a prospective study. Int J Behav Nutr Phys Act.

[41] Ogden J, Reynolds R, Smith A (2006). Expanding the concept of parental control: a role for overt and covert control in children’s snacking behaviour?. Appetite.

[42] Haycraft EL, Blissett JM (2008). Maternal and paternal controlling feeding practices: reliability and relationships with BMI. Obesity.

[43] Farrow CV, Blissett JM (2005). Is maternal psychopathology related to obesigenic feeding practices at 1 year?. Obes Res.

[44] Wasser H, Bentley M, Borja J, Goldman BD, Thompson A, Slining M (2011). Infants perceived as ‘fussy’ are more likely to receive complementary foods before 4 months. Pediatrics.

[45] Brown A, Rowan H. Maternal and infant factors associated with reasons for introducing solid foods. Matern Child Nutr. 2015. 10.1111/mcn.12166 PMC6860142 25721759

[46] World Health Organization, World Health Organization. Complementary feeding: report of the global consultation, and summary of guiding principles for complementary feeding of the breastfed child.

[47] Sachs M (2011). Baby-led weaning and current UK recommendations–are they compatible?. Maternal & child nutrition.

